# The Results of 45 Years of Atmospheric Corrosion Study in the Czech Republic

**DOI:** 10.3390/ma10040394

**Published:** 2017-04-07

**Authors:** Katerina Kreislova, Dagmar Knotkova

**Affiliations:** SVUOM Ltd., U Mestanskeho pivovaru 934, 170 00 Prague 7, Czech Republic; info@svuom.cz

**Keywords:** atmospheric corrosivity, atmospheric test exposure, yearly mass loss, long-term corrosion rate, structural metals

## Abstract

Atmospheric corrosion poses a significant problem with regard to destruction of various materials, especially metals. Observations made over the past decades suggest that the world’s climate is changing. Besides global warming, there are also changes in other parameters. For example, average annual precipitation increased by nearly 10% over the course of the 20th century. In Europe, the most significant change, from the atmospheric corrosion point of view, was an increase in SO_2_ pollution in the 1970s through the 1980s and a subsequent decrease in this same industrial air pollution and an increase in other types of air pollution, which created a so-called multi-pollutant atmospheric environment. Exposed metals react to such changes immediately, even if corrosion attack started in high corrosive atmospheres. This paper presents a complex evaluation of the effect of air pollution and other environmental parameters and verification of dose/response equations for conditions in the Czech Republic.

## 1. Introduction

Among several factors affecting the service life of metal structures, roofs, cladding, etc., atmospheric corrosion is recognized to be one of the major risks which impair the performance of constructions, resulting in huge economic and societal losses. Corrosion life prediction provides a key technology for the optimum selection of materials and/or coatings for such constructions. Corrosion scientists in many countries worldwide have carried out exposure tests to investigate the effects of the environment on corrosion rates. An evaluation period based on field tests usually takes 10–20 years.

**Figure 1:**
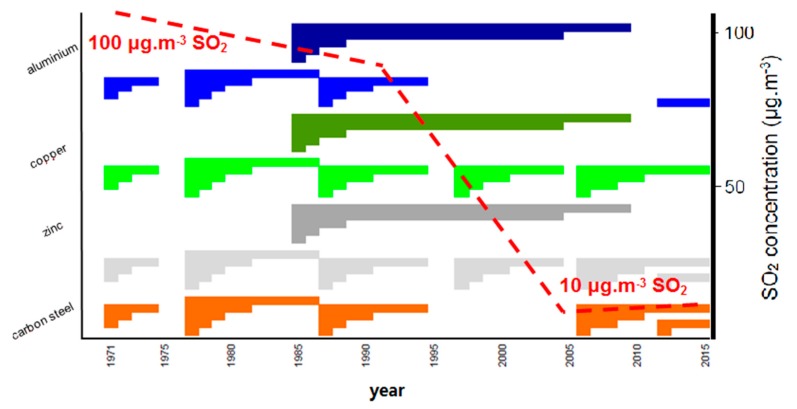
Scheme of exposure programs on atmospheric test sites during years 1970–2015.

In the Czech Republic (former Czechoslovakia) atmospheric corrosion has been studied since 1950 in the State research institute of material protection (SVUOM) [1,2,3]. Several exposure programs, both national and international, of structural metal atmospheric corrosion have been conducted at Czech atmospheric test sites since the 1970s, including on-site measurement of environmental data. As the Czech Republic is a relatively small country located in Central Europe, the most significant varying effect on the atmospheric corrosivity is imposed by air pollution, primarily that caused by industrial sources. In these repeated exposure programs, the standard flat samples of carbon steel, zinc, weathering steel, copper and aluminum were evaluated in intervals 1, 2, 3, 4, 5, 8, 10, 15, 20 and 25 years—Figure 1 [4]. The program data covering the period from 1985 to 2005, during which a significant reduction of SO_2_ pollution occurred, show a practically immediate decrease in metals corrosion rate [5]. The exposure program carried between 2005 and 2015 was realized in a relatively steady multi-pollutant situation. In 2015, the last new exposure program was launched with planned withdrawals after 1, 2, 5 and 10 years.

## 2. Materials and Methods

### 2.1. Materials

During each exposure period the structural metals were exposed as 100 × 150 mm^2^ flat panels on racks at an 45° to the horizontal, facing south:

(a) Carbon steel 1.0338 according to EN 10130 (C < 0.08%, P < 0.03%, Mn < 0.40%, S < 0.03%) [6];
(b) Weathering steel S 355JW Atmofix according to EN 10025-5 [7];
(c) Zinc (98.5%);
(d) Copper (99.8%);
(e) Aluminum (99.99%).

Triplicate panels in each withdrawal were treated with a standard procedure—degreased, rinsed by water and dried before exposure.

### 2.2. Atmospheric Test Sites

**Figure 2:**
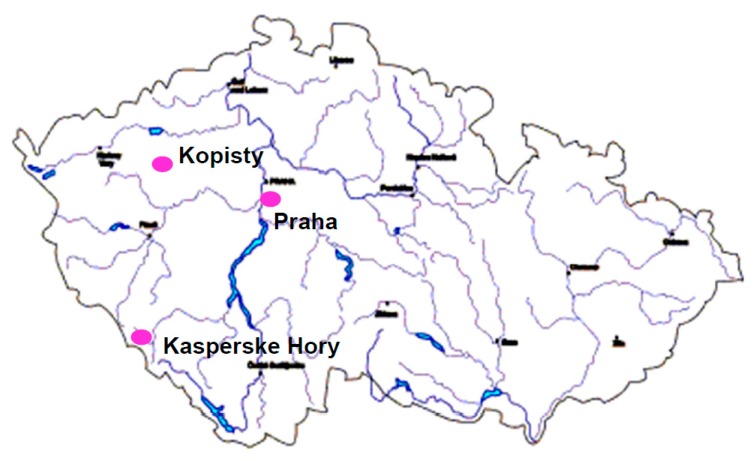
Atmospheric test sites in the Czech Republic.

Test sites are performed according to CSN 03 8110 [8] equivalent to ISO 8565 [9]. Over decades the network of atmospheric test sites changed. The test sites located in the current Slovak Republic have not been performed since 2000 (Hurbanovo, Lomnicky stit); some heavy industrial test site was closed too (Usti nad Labem, Czech Republic); in some specific exposure programs other test sites were involved (Ostrava, Telc, Horomerice, etc.), but there are 3 test sites that have been exploited for 45 years in the same locations (Figure 2):

(a) Prague—Urban atmospheric environment;
(b) Kopisty—Industrial atmospheric environment;
(c) Kasperske Hory—Rural atmospheric environment.

The environmental parameters significant for atmospheric corrosion have been measured at each test site during this period on a daily or monthly basis (temperature, relative humidity, precipitation, SO_2_ pollution, NO*_x_* pollution, pH of precipitation, chemical composition of precipitation, etc.) according to CSN 03 8110, respectively ISO 8565 and ISO 9225 [10] and statistically treated as annual average values.

## 3. Results

### 3.1. Environmental Data

**Figure 3:**
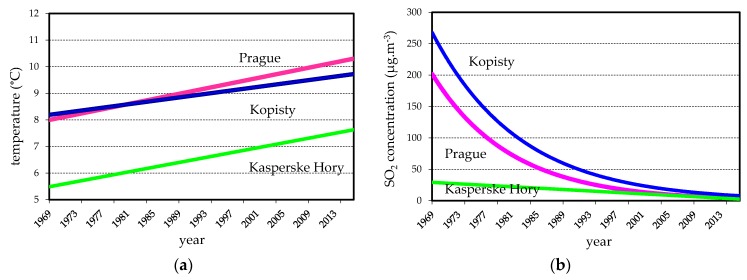
Trends in yearly average value of temperature (**a**) and SO_2_ pollution (**b**).

During 45-years the majority of climate parameters on these 3 test sites are stable with exception of yearly average temperature which increased (Figure 3a). During 1961–2010 average annual temperature increased for 0.7 °C and the annual amount of precipitation increased for 7% in North Bohemia region (test site Kopisty). In city Prague the thermal urban island effect is more evident. Estimated TOW is classified as τ_4_ for all test sites in the CR, e.g., for the Prague test site, the average TOW measured in period 1970–1980 was 3740 h·a^−1^.

When combined with climatic effects, air pollution can cause substantial deterioration of metallic materials. Sulphur dioxide and chlorides are the two dominant pollutants influencing atmospheric corrosion. The environmental parameters were relatively stable during the 45-year period, except SO_2_ air pollution and pH of precipitation which followed the changes in air pollution of SO_2_. The minor changes are observed in average annual temperature as opposed to very significant changes in average annual SO_2_ pollution—Table 1 and Figure 3b [4]. 

In the years before 1990, the test sites Prague and Kopisty were affected by air pollution from local heating and industrial sources; i.e., mainly by SO_2_. The first reports about the negative effect of the high concentration of SO_2_ emission appeared in the then Czechoslovakia as early as the second half of the 1940s, a trend comparable to the other highly industrialized countries/regions. Sulphur dioxide SO_2_ had the greatest influence on atmospheric corrosion rates in the 1970s to 1980s. Installation of desulphurization units at industrial sources, restructuring of industry and other large sources closing resulted in reduced SO_2_ emission in the CR within a relatively short period of 1994 to 2004 [11]. Afterwards, reduction of SO_2_ emission continued but not so intensively. Since 2010, a minimal difference in SO_2_ air pollution among rural, urban and industrial test sites has been measured. This situation is observed at all atmospheric test sites, mainly at the industrial site Kopisty and at the urban site Prague. Together with this acidic air pollution decrease the pH value of precipitation increase slightly from 5.0 to 6.0.

Measurement of nitrogen oxides (NO*_x_*) air pollution started only later, namely in the middle of 1980s. The effect of NO*_x_* on atmospheric corrosion are still only assumed above 30 μg·m^−3^ [12] and long-term measured data from Prague and Kopisty show that average values oscillate around this value. The ratio SO_2_/NO*_x_* is ca. 0.5 since 2000. NO*_x_* reacts with air to form strong corrosive gaseous nitrogen acid HNO_3_ but its average concentrations measured at the Prague and Kopisty test sites in the years 2003 to 2015 were 0.82 μg·m^−3^ and 0.57 μg·m^−3^, respectively.

Prior to the 1950s, atmospheric corrosion caused by airborne salinity was limited to coastal areas. However, in the 1970s the widespread use of de-icing materials on roadways led to a serious corrosion problem in the snowbelt regions/countries. Field tests have shown that approx. 40 wt. % of NaCl de-icing salt applied to roadways is carried by the air to be deposited within 100 m of treated road and may accumulate on their side and become part of dust. Cl^−^ deposition was measured in 1980s at the Prague test site and average annual value was 4.8 mg·m^−2^·d^−1^. The chloride deposition rate has been measured at the atmospheric test sites Prague and Kopisty according to EN ISO 9225 since 2016. The chloride deposition at the Prague atmospheric test site was approximately twice (ca. 3.0 mg·m^−2^·d^−1^) the values identified at the industrial atmospheric test site Kopisty (ca. 1.3 mg·m^−2^·d^−1^). These values are at the background level S_0_ according to ISO 9223.

### 3.2. Corrosion Data

After exposure, all panels were visually examined before determining gravimetric corrosion mass losses by removing corrosion product layers according to ISO 8407 [13] for each metal. Weight loss was calculated by area and time.

**Figure 4:**
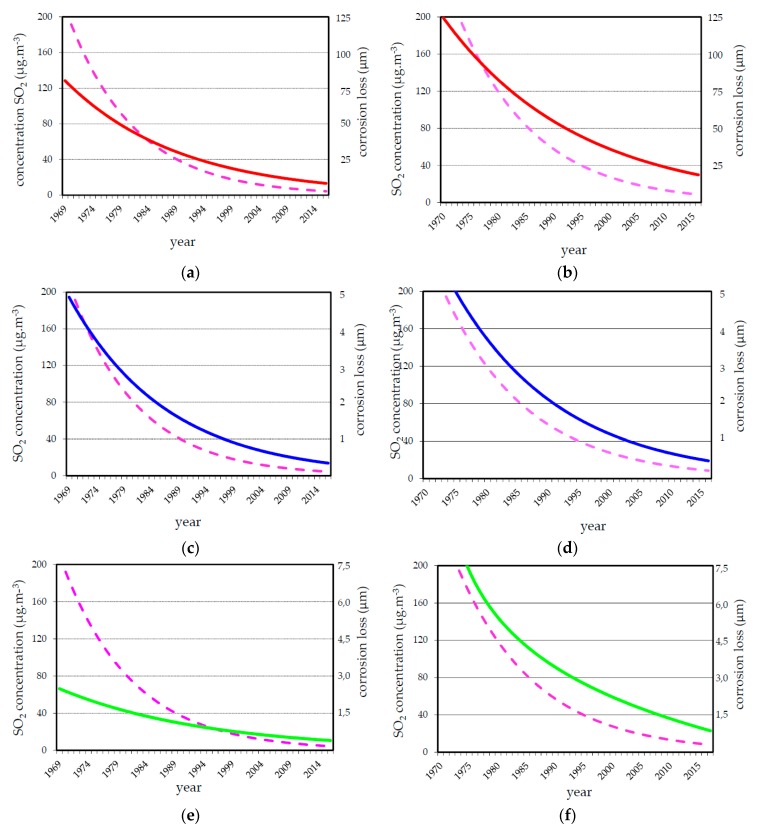
Trend analysis in annual corrosion losses (bolt line) and SO_2_ pollution (dashed line) at Prague (**a**,**c**,**e**) and Kopisty (**b**,**d**,**f**) test sites in different exposure periods for carbon steel (**a**,**b**); zinc (**c**,**d**); and copper (**e**,**f**).

In 45 years many repeated one-year exposure of structural metals had been performed (see Figure 1). As SO_2_ pollution level changed during this period the one-year corrosion loss decreased too. There was some varying in corrosion data due to not stable climate conditions (warm or cold summer, warm or cold winter, rainy season, etc.). The trend analysis based on statistic treatment of measured values undertaken within a formal regression analysis of one-year corrosion loss of carbon steel, zinc and copper panels are shown in Figure 4 (bolt line) along with the trend of SO_2_ decrease (dashed line) in the Prague and Kopisty test sites; there are fewer corrosion data available for Kasperske Hory test site throughout this entire period. These trend analyses based on statistic treatment of measured values show that the atmospheric corrosion of carbon steel and copper is more strongly affected by SO_2_ than the atmospheric corrosion of zinc.

These databases can be used to verify models of atmospheric corrosion. The functions that allow calculating corrosion loss based on environmental data are derived to predict the corrosion behavior of metals in the future.

## 4. Discussion

Corrosion losses r_corr_ estimated after one year of exposure on carbon steel, zinc and copper are used to classified corrosivity at the test sites in accordance with standard ISO 9223 [14]. Local climate is defined as conditions prevailing within the radius of an object/locality up to 1000 m. Local climate and the pollutant content provide a basis for the determination of an atmospheric corrosivity category.

The corrosion growth rate has always been a function of time. For long- term exposure predictions this ISO 9224 [15] equation can be used:

D = r_corr_·t^b^

where t is the exposure time in years, r_corr_ is the attack experienced in the first year in mm or μm, b is the metal-environment specific time exponent which is different for each metal.

The changes in average air temperature and mainly evident changes in amount of precipitation occurred since 2000 more frequently may affect the short-time, one-year corrosion data but they are not yet evident for long-term corrosion behavior of structure metals. But for application of long-term corrosion model it is necessary to use r_corr_ as average value for some last exposures or from period with relatively average climate parameters. For the statistical treatment of this extensive database of 45 years of atmospheric corrosion field exposure measured values and the trend analyses resulting therefrom, three periods may be created: two periods with relatively stable environmental conditions (the years 1970–1994 and the years 2004–2016) and a transmission period 1994–2004 with quickly changing air pollution level. The average data for these periods are given in Table 2.

A number of damage functions or dose-response equation, which are compared to the atmospheric corrosion of metals using environmental parameters applicable in Europe, were derived in several studies:

(a) ISOCORRAG-ISO 9223 and ISO 9224 [16];
(b) UN ECE ICP Materials [17];
(c) EU project EVK4-CT 2001-00044 MULTI-ASSESS [18].

**Figure 5:**
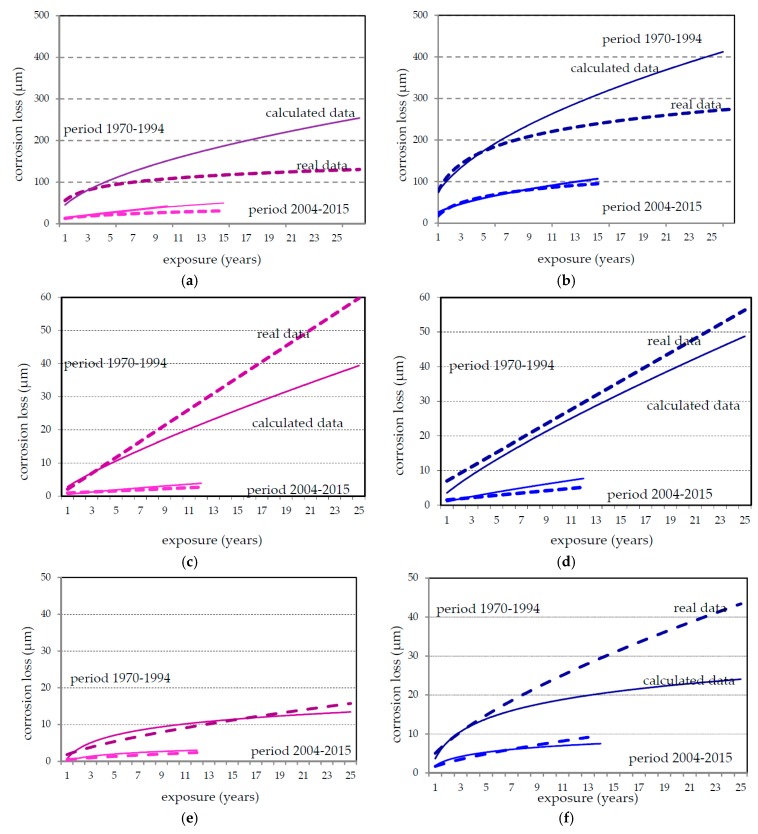
Trend analysis in long-term corrosion losses at Prague (**a**,**c**,**e**) and Kopisty (**b**,**d**,**f**) test sites in different exposure periods for carbon steel (**a**,**b**); zinc (**c**,**d**); and copper (**e**,**f**).

All of these dose-response equations are derived from field exposure results obtained in the years 1986–2004 where the SO_2_ level was relative high at the urban test sites and very high at the industrial test sites. The data from all exposure programs performed in the Czech Republic (or former Czechoslovakia) were used to verify these prediction models. The best fitting was established for ISO 9224 equations [19]. With regard to long-term corrosion data, comparison of the results of the Czech national programs performed in the years 1970–1990 and 2005–2015 was completed for Prague and Kopisty test sites—Figure 5.

A survey of long-term exposed materials (weathering steel structures, zinc coating on galvanized structures, copper roofs, aluminum products) was also included in these data [20].

The differences between predicted (calculated) values and real (estimated) corrosion loss was caused the fact that dose-response functions were derived from database which does not include these corrosion values from high pollution years. The ISO prediction model for one-year atmospheric corrosion corresponds relatively well with real (determined) values but in exposures with SO_2_ > 90 mg·m^−2^·d^−1^ calculated values are significantly different from determined corrosion loss; in case of the long-term prediction model the fitting of real (determined) to calculated values is better for atmospheres with lower SO_2_ pollution. The first results show it would be better to divide the dose-response functions according to limit pollution values to obtain more accurate prediction models. To enhance atmospheric corrosion prediction, interaction between all air pollutants should be considered in current multi-pollutant environmental condition, especially for zinc and copper.

**Figure 6:**
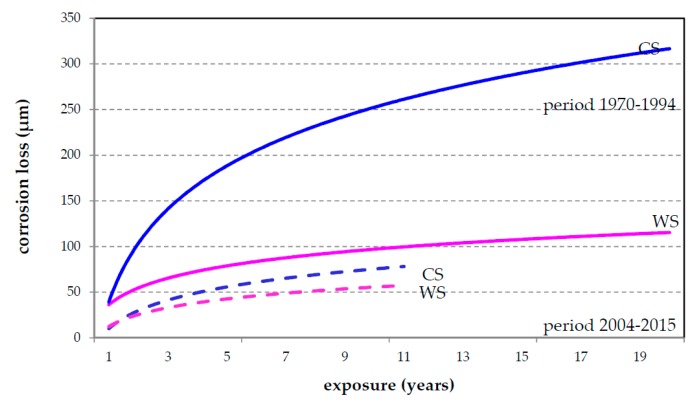
Comparison of corrosion loss of carbon (CS) steel and weathering steel (WS) at Kopisty.

Changing environmental conditions also affected significant structural material of weathering steel. This effect has been evident since the first year of exposure, but a decreasing corrosion rate is more important for long-term data. The protective effect of patina is more evident for atmospheres with high SO_2_. Only 8 years of data are available from the Czech test sites for this material with regard to the current environmental situation. Comparison of the long-term trends of the atmospheric corrosion of carbon and weathering steel in industrial locality Kopisty shows that the corrosion rate of weathering steel decreases less than that of carbon steel (Figure 6). The corrosion loss of weathering steel, respectively the protective effect of patina, is more significantly affected by climate parameters in environments without salinity.

The corrosion rates shown in ISO 9223 and ISO 9224 were estimated as uniform corrosion, but aluminum corrosion manifests itself rather as pitting corrosion. The rate of the deepening of pits formed in atmospheric environments decreases with time. Studies showed that the deepening rate of pits follows the equation [21]:

*d* = *k·t^⅓^*

where *d* is the depth of the pit, *t* is the time, and *k* is a constant depending on the alloy and the service conditions (nature of the alloy, temperature, atmospheric corrosivity category, etc.).

**Figure 7:**
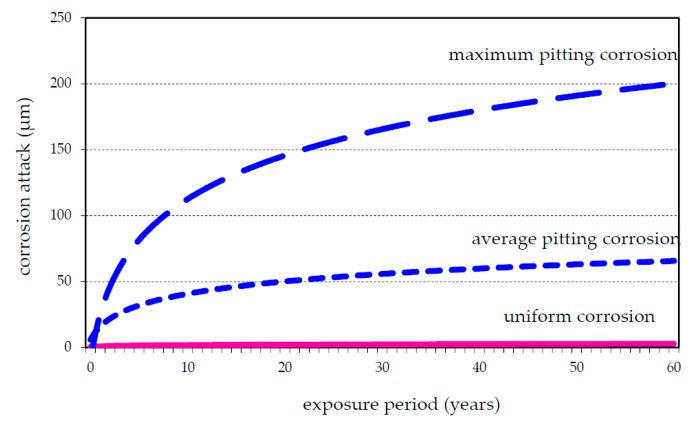
Uniform and pitting corrosion of long-term exposed aluminum in CR.

Maximum pit depth is a better indicator of potential damage, but this characteristic cannot be evaluated after the first year of exposure. Evaluation of long-term exposed aluminum materials shows that the largest increasing of pit depth occurred within 5 years. Average rate of the long-term pitting corrosion of aluminum exposed in CR atmospheric environment is 1.5 μm·a^−1^ (Figure 7). This value corresponds to published data. From experimentally evaluated data, constant ***k_ave_*** for CR atmospheres was estimated as 18 for the average rate of pitting corrosion. The same approach is defined for maximum pitting corrosion rate and constant *k**_max_*** is 50.

## 5. Conclusions

The short- and mainly long-term atmospheric corrosion tests of structural metals provide significant information for improving prediction models of the atmospheric corrosion. Data mining, applying to corrosion, represents the selection tools available to quantify a response that a given metal will give in a given environment and the effect that environment changes will have on that metal. A large database of environmental and corrosion data available for the Czech test sites allows us to follow the trends and effects of air pollution changes on the atmospheric corrosion of structural metals.

The main changes in atmospheric corrosion rate were caused by SO_2_ deposition. The obtained results confirm the significant dependence of atmospheric corrosion of all exposed metals on SO_2_ air pollution and show that carbon steel atmospheric corrosion depends on SO_2_ pollution level much more than zinc and copper atmospheric corrosion. The reduction of SO_2_ has an impact on both short- and long-term corrosion rate. The exposure programs continue as one-year and long-term programs with extended measurement of the other types of pollution because, in past decades, the impact of SO_2_ has markedly prevailed over any other pollutants.

## Figures and Tables

**Table 1:** Selected average annual environmental parameters at test sites.

Test Site	Year	T (°C)	RH (%)	SO_2_ (µg·m^−3^)	NO*_x_* (µg·m^−3^)	Rain (mm)	pH of Precipitation
Prague	1970	8.6	76	92	-	490	-
	1980	7.4	77	99	-	653	-
	1990	10.1	74	56	34	468	5.4
	2000	10.2	72	11	23	498	5.7
	2016	10.0	73	5	28	561	6.0
Kopisty	1970	8.1	76	182	-	618	-
	1980	7.6	75	145	-	520	-
	1990	9.7	71	67	32	398	4.9
	2000	10.2	76	16	28	486	4.7
	2016	9.9	76	18	23	567	6.0
Kasperske	1970	5.4	79	23	-	637	-
Hory	1980	5.4	80	30	-	811	-
	1990	7.0	78	26	9	722	4.9
	2000	7.8	76	8	9	798	5.4
	2016	8.7	74	11	24	841	6.0

**Table 2:** The comparison of estimated and predicted long-term corrosion rate.

Test Site	Period	Environmental Data			Corrosion Rate (μm·a^−1^)					
		T (°C)	RV (%)	SO_2_ (µg·m^−3^)	Carbon Steel		Zinc		Copper	
					Real	Cal	Real	Cal	Real	Cal
Prague	1970–1994	8.8	77	87.6	16.1	15.1	2.82	2.30	1.30	0.86
	2004–2015	10.1	73	7.3	2.6	4.0	0.13	0.45	0.22	0.23
Kopisty	1970–1994	8.7	74	119.2	21.4	25.0	3.66	2.66	2.26	2.35
	2004–2015	9.3	78	14.2	9.3	8.6	0.33	0.89	0.51	0.73

## References

[1] Barton K., Knotkova D., Spanily J. (1973). Errechnung der Geschwindigkeit der atmospharischen Korrosion nach meteorologischen und Luftverunreinigungswerten. Vortrage des Wissenschaftlichen Kollquims Bearbeitung meteorologischer Werte in Zusammenhang mit der Erforschung der Atmospharischen Korrosion.

[2] Barton K. (1976). Protection against Atmospheric Corrosion.

[3] Knotkova D., Bosek B., Vlckova J. (1974). Corrosion Aggressively of model regions of Czechoslovakia. Corrosion in Natural Environments.

[4] Kreislova K., Geiplova H., Majtas D. Long-term study of structural metals’ atmospheric corrosion in the Czech Republic. Proceedings of the EUROCORR 2016.

[5] Kreislova K., Knotkova D. Corrosion behaviour of structural metals in respect to long-term changes in the atmospheric environment. Proceedings of the EUROCORR 2011.

[6] (2006). Cold Rolled Low Carbon Steel Flat Products for Cold Forming—Technical Delivery Conditions.

[7] (2004). Hot Rolled Products of Structural Steels—Part 5: Technical Delivery Conditions for Structural Steels with Improved Atmospheric Corrosion Resistance.

[8] (1978). Protection against Corrosion—Atmospheric Test Stations—General Requirements (Czech Technical Standard).

[9] (2011). Metals and Alloys—Atmospheric Corrosion Testing—General Requirements.

[10] (2012). Corrosion of Metals and Alloys—Corrosivity of Atmospheres—Measurement of Environmental Parameters Affecting Corrosivity of Atmospheres.

[11] Kreislova K., Knotkova D., Tidblad J., Henriksen J. Trends in corrosivity of atmosphere and material deterioration in Europe region in period 1987–2001. Proceedings of the Abstracts of Conference Acid Rain 2005.

[12] Arroyave C., Morcillo M. (1995). The effect of nitrogen oxides in atmospheric corrosion of metals. Corros. Sci..

[13] (2009). Corrosion of Metals and Alloys—Removal of Corrosion Products from Corrosion Test Specimens.

[14] (2012). Corrosion of Metals and Alloys—Corrosivity of Atmospheres—Classification, Determination and Estimation.

[15] (2012). Corrosion of Metals and Alloys—Corrosivity of Atmospheres—Guiding Values for Corrosivity Categories.

[16] Knotkova D., Kreislova K., Dean W.S. (2010). ISOCORRAG—International Atmospheric Exposure Program: Summary of Results.

[17] Kucera V., Tidblad J., Kreislova K., Knotkova D., Faller M., Reiss D., Snethlage R., Yates T., Henriksen J., Schreiner M. (2007). UN/ECE ICP Materials dose-response functions for the multi-pollutant situation. Water Air Soil Pollut. Focus.

[18] Watt J., Tidblad J., Kucera V., Hamilton R. (2009). The Effect of Air Pollution on Cultural Heritage.

[19] Geiplova H., Kreislova K., Mindos L., Turek L. (2016). Evaluation of long-term exposed structures and their maintenance. Corrosion and Surface Treatment in Industry. Mater. Sci. Forum.

[20] Hatch J.E. (1984). Aluminium—Properties and Physical Metallurgy.

[21] Vargel C. (2004). Corrosion of Aluminium.

